# Supplementary material for: Large-scale design and refinement of stable proteins using sequence-only models
Source: PLoS One. 2022 Mar 14;17(3):e0265020. doi: 10.1371/journal.pone.0265020 (PMC8920274; doi:10.1371/journal.pone.0265020)
Supplement: Attachment — Submitted filename: PLOS rebuttal letter.rtf [file pone.0265020.s040.rtf]

We include below comments from the three reviewers when we submitted an earlier version of the manuscript to PLOS Computational Biology. We are grateful for their incredibly thorough reading of this large manuscript. In nearly all cases, we believe we were able to address their questions or concerns. In the remaining few cases, we believe that the suggested change is far outside the scope of the paper. Our responses to the comments and questions are in-line, in bold.

Thank you,

Jed Singer on behalf of all authors.


Reviewer #1: The manuscript has described a data-driven high-throughput process to automate the design of stable proteins, with a potential to generalize to protein engineering for other properties.  It is built on a previous data-collection platform (ref. 20) consisting of parallel oligo library synthesis, yeast surface display,  next-generation sequencing, and Aquarium lab automation software (aquarium.bio).  It is equipped with two neural network models for the design and refinement of sequences that can iteratively get feedback from and send feedback to the data collection component.  

Specifically, an "evaluator" model (EM), a convolutional neural network, was introduced for sequence-to-stability (and secondary structure) prediction.  Besides the two tasks of stability and secondary structure prediction for designed proteins with experimental stability measurements, an additional loss term to train the model was to let natural proteins be predicted more stable than scrambled. Another "generator" model (GM) started with a secondary structure sequence and predicted an amino acid sequence of he same length (an analogy to natural language translation); and the GM used a recurrent neural network encoder/decoder architecture with attention.  

The two models were analyzed comprehensively in conjunction with the experimental data (whether  source data to train models or measurements of model-generated designs).  Many expert questions were asked and careful analyses were performed to assess models' power, limitation, and learned knowledge, which provided many insights for protein engineers and model developers.  The EM was found to predict stability score well for expert and GM-designed sequences; and due to its speed, could afford a greedy method (5-round beam search) and filter promising 1-5 mutations from starting designs. One stable design was structurally predicted by trRosetta and structurally determined / verified by X-ray crystallography. 

This is a very exciting study: a powerful data-driven protein engineering process with potentials for other design properties; and many domain knowledge-inspired tailoring and interrogating the models that drive the designs.  Please consider addressing the following comments which could potentially improve the manuscript further, especially EM model generalizability (comment 1) and GM model capability to generate novel sequences (comment 6) 


1. The training and test data for the EM were randomly split, thus the model generalizability cannot be assessed by the test set performance such as Figure 2C.  Please consider splitting the test set into bins of various maximum sequence identity (MSI) ranges compared to the training set, make a figure of stability prediction performances (such as r^2) over all MSI bins, and put special attention to the bin with MSI less than 30% (non-homologs to training sequences).  If the number of non-homologs in the current test set is too low to support a statistical meaningful conclusion, please consider re-split the data set for a test set of enough non-homologs. 

Besides generalizability in sequence, that in protein classes such as topologies, design approaches, and sizes is also worth checking. Figure S9 dealt with it but it considered 27 combinations of all 3 class types and only left one out as the test set each time. The consequence was that, for instance, when the test set was a triple helix HHH topology from library 3, the training set included the same topology from library 1; so it remained unclear whether the EM can predict stability well for HHH if such topology was not in the training set.  Therefore, a marginal split rather than the joint split is recommended; and attentions should be paid that EEHEE includes a sequence segment of EHEE and 1a4b may include a sequence segment of 1a3b. 

To address concerns about similarities between classes of proteins, we added BLAST analyses (lines 151-158) in which we found each protein’s closest homolog from among the 26 other classes; we found relatively low bit scores and percent identities. We also found no correlation between model error and either of these quantities, suggesting that the EM was not leveraging what homology there was between classes.

2. Again for EM, Figure S10 has a visible pattern across all panels: many dots lined up a vertical line at the same predicted score (x-axis value), indicating that many designs of various stability scores were predicted by EM to possess exactly the same stability across all 14 perturbations.  Any explanation? What was the significance of the same predicted stability score observed on those vertical lines? Was it near the training average?  

We were unable to figure out a good explanation for this pattern (which is also apparent in Fig. 2). We have added an acknowledgment of it, and the fact that neural networks can make finding answers to such questions difficult, in lines 180-183.

3. The training corpus of the models are extremely important to their powers. 

So that the EM can be as accurate as needed when exploring sequence space outside of the training sequences, how diverse was the training corpus? Any chance to quantify or visualize the diversity and compare that to all the neutral sequences, such as showing the (t-SNE low-dimensional) embeddings of training corpus and natural proteins from protein language modes such as UniRep, Prot_Bert or ProtTrans?

So that the EM can be as accurate as needed when refining designs by introducing few mutations, what was the "granularity" of the training corpus? Any chance to quantify or visualize it? 

We agree that these are interesting and important questions. However, this manuscript is already very long, and there isn’t a single canonical approach that would address them to everyone’s satisfaction. We do make the data available, and suggest that people who seek answers to these or related questions may be in the best position to find their answers to their specification.

4. The loss to train EM was the weighted sum of three terms and each term was again the weighted sum of terms.  How were the particular weights determined and how did they affect EM performance? 

We now note (lines 823-825) that the weights were chosen early in development because they worked well but that model performance is fairly robust to changes in those weights.

5. For GM, there are a couple inconsistencies in descriptions.  The GM training data was described as a subset of the training data for EM (Line 281) but then described as "unlike EM we did not hold out any designs for testing" (so EM training data is the subset of GM?).  Maybe the truth was mixing both:  used the EM training and test data less unstable designs? Similarly, Line 257 said that perplexity was for the test set then why did Line 268 interpret the perplexity = 1.1 as generated sequences being similar to "training" sequences rather than ground-truth sequences of the test set? 

Thank you for highlighting this confusion; we have clarified the indicated section.

6. It was stated that GM could generate "novel" sequences but more data is needed to back the statement.  Rather, among 993 expressed designs, only 275 and 120 were with MSI below 95% and 75%, respectively, compared to the training set (Lines 276 - 279).  Not to mention that 95% and 75% are very lenient thresholds. How many were with MSI below 30%, i.e., non-homologous to training sequences?  And among them how many were stable? Figure S25 seemed to indicate all or nearly all the 1000 top designs were of MSI above 30%, thus homologs to training sequences. Then what about testing a few top designs whose MSIs were below 30%?  

We have highlighted (lines 272-275) that the GM tends to produce sequences very similar to those in the training set, but that it can be used to find less-similar sequences (lines 432-433, 531-534). For Corpus B we did not apply such a constraint, however, and it is true that almost none had <30% identity.

7. The GM formulation of natural language translation (from secondary structure sequence to amino acid sequence of the same length) had no input of the relative orientations of secondary structures or other tertiary information that drives stability.  How does this limit protein design in general and that for for stability?  BTW it was a nice idea to weigh training sequences proportional to their stability. 

At this point, the GM is only suitable for the rapid generation of stable, or stabilizable, mini-protein sequences. We make explicit its current uses in lines 276-278, 529-534).

8. The current models are focused on mini proteins of length 28-65 (mean 55). Is there any challenge in extending the framework to longer sequences? Is the bottleneck in the data, the models (especially RNN for GM), and more? Please consider a short discussion. 

Both; we have added a short discussion in lines 561-567.

9. Why were cysteines excluded from sequence generation by GM? Is it to prevent disulfide bonds as a cheap shortcut to increase stability? Any negative impact of not considering cysteines in GM?

The stability assay is incompatible with disulfide bonds, see lines 299-301.

Reviewer #2: Review of “Large-scale design and refinement of stable proteins using sequence-only models” 
PLOS Computational Biology.

Description of the work: the authors apply a high-throughput assay for collecting large datasets of protease susceptibility data for mini-proteins (maximum of 65 residues in length). New datasets, along with other previously published sets, are used to train two neural network models: a predictor of a _stability score_ and a generative model of primary sequences of mini-proteins. The models are trained encoding primary sequences as sparse logic vectors and using also topology (secondary structure) information. The main findings are that designed mini-proteins are usually more stable than randomly perturbed sequences according to the defined stability score and that the ML+assay pipeline can successfully generate and test large numbers of these mini-proteins, and designs can have their stability scores improved iteratively with guided single mutations. 

General comments: The combination of machine learning and high-throughput experimentation using an automated pipeline is an important step in the direction of improving scalability of protein design efforts. The ability to design new mini-proteins and successfully refine them iteratively indicates that with suitable assays and large investments in automated platforms, large-scale improvement of protein properties could become a standard tool for researchers. Despite the large amount of work presented, my difficulty is with the significance of the manuscript to the field. Assay, stability scores and some of the datasets were previously published. The prediction of stability with neural networks and the refinement with mutations are interesting but not novel. I was confused by the fact that the models are presented as sequence-only, although both used topology data (secondary structure). In the current form the manuscript is of limited interest. Here are some possible
directions that could be attempted:

We have clarified that “sequence-only” refers to the models’ inputs, not to the information used during training.

1)  All the designs produced are scaffolds without function. There could have been attempts to design a function in large-scale and evaluate its impact on stability. 

While true, this would have been a substantially larger undertaking (though it has been underway for several years now!)

2) Since the authors have the possibility of generating large quantities of experimental data, a comparison with different alternative models (closely related previously published work) would increase the significance of this work, allowing researchers to see which directions are more fruitiful when choosing between alternative modeling techniques. Some possible interesting research that has worked with stability prediction and design refinement are:

[1] Li, Guangyue, Youcai Qin, Nicolas T. Fontaine, Matthieu Ng Fuk Chong, Miguel A. Maria-Solano, Ferran Feixas, Xavier F. Cadet, et al. 2020. "Machine Learning Enables Selection of Epistatic Enzyme Mutants for Stability Against Unfolding and Detrimental Aggregation." ChemBioChem, November, cbic.202000612.

[2] Laimer, Josef, Heidi Hofer, Marko Fritz, Stefan Wegenkittl, and Peter Lackner. 2015. "MAESTRO - Multi Agent Stability Prediction upon Point Mutations." BMC Bioinformatics 16 (1): 116. 

[3] Wang, Huilin, Liubin Feng, Ziding Zhang, Geoffrey I. Webb, Donghai Lin, and Jiangning Song. 2016. "Crysalis: An Integrated Server for Computational Analysis and Design of Protein Crystallization." Scientific Reports 6 (1): 21383.

The last reference is particularly interesting since it links stability with crystalizability. Such comparisons with alternative modeling techniques help catalyze more work in the field, since they create dialog between research efforts.

We have included the first two references in the introduction (lines 39-43); while we agree that the third is quite interesting we struggled to find a connection to our work direct enough to demand mention.

3) An interesting question is whether the observed prediction performance is due to more training data available or to deeper and more complex models. A comparison with alternative predictors would allow us to better answer the question, for the small proteins in this study, how different levels of model complexity (e.g feed-forward models) impact prediction when data availability is not a hurdle anymore. This could be addressed with the high-throughput pipeline the authors have developed. It would also add a baseline model to compare the gains provided by the EM model architecture.

We agree that these are interesting questions; answering them thoroughly would add considerably to the manuscript’s bulk, however, and we think they are out of scope.

On the technical side of the current manuscript I have the following specific comments:

1) The GM (Pg12.Lns262-268) had the benefit of conditioning on topology (secondary structure), while the basic RNN model did not. It seems that a fairer comparison would have the basic RNN model also make use of topology information. Without this, it is less clear if the gain in perplexity observed (1.1 vs. 1.8) is due to model differences or differences in what information is available to the model.

The topology information is exactly what the GM gains over a basic RNN. We have clarified this point (lines 305-308).

2) From the text is unclear whether the GM does not generate cysteines or simply that cysteines were not accounted for in the perplexity computation. It would be important to clarify this point, since without this information is difficult to understand if the authors are assuming that cysteines are not important for stability (and should not be generated) or if cysteines (due to their low probability in the language model) preclude a good validation of the model using perplexity.

We have clarified that C is not in the GM’s vocabulary (lines 299-301) because of incompatibility with the stability assay.

3) It is common to think of sequence-based models as based on primary sequence information (logical encodings, biophysical properties, amino acid composition), as introduced in the Abstract: "Here we report a neural network model that predicts protein stability based only on sequences of amino acids...". However, given that topology is necessary in both models, the authors could consider rewording _sequence-only_ to reflect the fact that secondary structure (topology) predictions are essential in predicting stability and in successfully generating stable mini-designs.

We change “based on” to “given” to indicate that the trained model requires only primary sequences as input.

4) Given the rich amount of data generated, it is currently difficult to follow which datasets are being used at each step and to keep track of the number of designs in each set. In "Materials and Methods" authors explained how datasets were divided and assigned to “Libraries”. However, the Library terminology is not used in the main text. Connecting the terminology of "Materials and Methods" with the main text could help the reader in keeping track of the different rounds of simulation. One way the manuscript could be improved is by denoting each dataset by a letter A, B, C, D, ... and referring to them by these letters in the main text. To illustrate and make clear for the authors the difficulties I had, here is one example of where identifying datasets more clearly could help (pg17.lns379-385):

To further examine how EM-guided refinement increases stability score, as well as to
demonstrate the feasibility of performing this refinement under a range of constraints,
we generated and assayed another set of 97,702 proteins, including three sets
representing refinements governed by increasingly stringent constraints. GM designs on
this chip were required to have less than 50% sequence identity with the most similar
expert-designed protein, and all refinements used a new version of the EM trained on
the union of the original training set and the new proteins described in previous sections.

Using a more systematic notation could improve clarity:
To further examine how EM-guided refinement increases stability score, as well as to
demonstrate the feasibility of performing this refinement under three types of constraints,
we generated and assayed another set of 97,702 proteins (Dataset D), including three sets
representing refinements governed by three increasingly stringent constraints (Datasets D.1, D.2 and D.3). Dataset D contains GM designs all assayed on a single chip, and were required to have less than 50% sequence identity with the most similar expert-designed protein from Dataset B. All refinements in Dataset D used a second version of the EM (EM-2) trained on
the union of the original training set (Dataset A) and the new proteins described in previous sections (Datasets C.1, C.2).

A second example (pg 31.lns800-801): "To estimate the parameters of the GM, we considered the same expert-designed proteins used to train the EM." It is unclear which designs were used, since the EM was trained more than once.  A more systematic notation can help clarify: "To estimate the parameters of the GM, we considered the same expert-designed proteins (Dataset A) used to train the EM-1."
The models can also benefit from such naming convention (e.g. EM-1, EM-2, GM-1, GM-2), since they were trained more than once (Pg17.Ln384; Pg31.808) with different datasets.

Yes, this was confusing! We have adopted a similar approach to the reviewer’s suggestion, and now refer to Corpus A (comprising Libraries 1-3), B, and C. We thought introducing proper nouns at the level of the reviewer’s suggestion might actually further confuse things, but we hope this compromise is clarifying.

5) Figure 1 shows small structures where the data is actually a primary sequence. The authors could consider not using structural representations as an illustration of primary sequences, since this can lead readers to think that the models require 3D structural data to function or that the models can generate 3D structures instead of primary sequences.

Good point, we have made the suggested change.

6) The authors made an effort to avoid similarity of topology and design methodology in some of the generated datasets when compared to other datasets. However, it was not clear from the text, if within the datasets used for the EM training, redundancy was controlled for (e.g. using BLAST sequence similarity). It seems that some datasets could be carrying designs with highly similar sequences. Since validation was performed with random examples, it might be interesting if the authors would consider checking and reporting sequence similarity within a single dataset.

As noted above, to address concerns about similarities between classes of proteins, we added BLAST analyses (lines 151-158) in which we found each protein’s closest homolog from among the 26 other classes; we found relatively low bit scores and percent identities. We also found no correlation between model error and either of these quantities, suggesting that the EM was not leveraging what homology there was between classes.

7) I would ask the authors to consider adding versions/builds for the different software packages used (Keras, TF, etc.). This will allow users to address future issues of backward (in)compatibility arising from the fast cycle of updates some of these packages have.

Absolutely; done (line 834).

Minor points:
1.minor) In some instances, the language in the manuscript is subjective and could be made clearer by giving counts and percentages. A more objective language will help the reader in evaluating the significance of the results. Some examples: Pg22.ln1: “several datasets” (authors could cite the actual number), Pg21.ln1: “reasonable accuracy” (authors could provide the percentage); Pg20.Ln482 “large numbers” (authors could provide the count of how many examples were generated). Pg18.Ln438 “with high accuracy” (a percentage could be given). Pg18.Ln435 “massively parallel” (authors could indicate number of sequences that can be tested simultaneously). Pg17.Lns388-390: “Refinement of expert-designed sequences...slight improvement stability (authors could provide a mean value), compared with a substantial decrease (authors could provide a mean value) to stability...”. Pg17.Ln380 “under a range of constraints” (authors could state the number of constraints).

We have added the requested clarifications (in cases where they were not already present in adjacent sentences).

2.minor) I would suggest that, in the manuscript, the authors consider not referring to the GM as the "second neural network", since this does not reflect the image or the caption in Fig 1.

Good point; fixed.

3.minor) It seems that dTERMen is currently missing from the image in Fig. 1 alongside with "expert scientist + Rosetta".

Fixed.

Reviewer #3: In this manuscript, the authors explored the idea of using large-scale experimental data for neural network training, and in turn, use the neural networks to generate new protein sequences to see if they achieve the training objective, which is stability (as revealed by protease resistance). The authors first used data from previously generated de novo proteins for training a predictive model that was able to capture the correlation with chymotrypsin/trypsin resistance, followed by using the model for stability improvement. Then the authors trained a generative model and setup an iterative refinement with the stability predictive model to improve the apparent protease stabilities. 

The amount of data that went into this paper and the care the authors took to analyze them is very impressive.  Although I recommended rejection (for reasons below), there are definitely very valuable lessons here. The large-scale experimental approach that the authors used here is certainly the right idea, but unfortunately the protease resistance is too crude of a measure. Given the unsatisfactory results, it is very difficult to recommend publication with high enthusiasm. 

Main issues:
(1) The fundamental question is whether a model that can translate a string of secondary structure into amino acid sequence has any significance. Perhaps the assumption was that since the Rosetta generated designs started out as blueprint topologies, a model that can quickly render a stable sequence for those topologies would somehow facilitate protein engineering. Unfortunately, as it has been shown by Dr. Baker's group, that the de novo proteins are highly relevant for developing binders for a wide range of targets is because of their 3D shapes. A sequence-only generator does not provide the same level of significance. 

We have highlighted that, at this point, the GM is solely a proof-of-concept for translation between secondary and primary structure, and a source of stable or stabilizable mini-proteins (lines 276-278).

(2) Data in figure 7 essentially undermine the entire study. What it revealed was the fact that the stability score (either computed or experimentally derived values) has almost no real predictive power for the proteins' stability when expressed and purified as recombinant proteins. This was already observed in a publication from Dr. Baker's group (Basanta, et. al. PNAS September 8, 2020 117 (36) 22135-22145). In the Basanta paper, they mentioned that perhaps the protease assay was not reliable for larger proteins. This manuscript seems to show that it is also not a good assay for small proteins of similar sizes to those from the originally Rocklin et al. paper (ref 20). It is commendable that the authors for this paper went the extra mile to solve a crystal structure. However, firstly, the structure does not follow the input 4 segment helical topology, and, secondly, the underlying mechanism that drives this 3-helix topology does not lend much credit to the generator model.
From the primary sequence for the crystalized protein, it is very obvious that the sequence folded into 3 different segments because there are 3 proline residues at the beginning of each of the helices. The generator model (GM) arguably provide the proper sequences for forming the helix, but we really don't know that; most of the residues used in the helical regions are residues of high helical propensity.  It is really not proven that the GM provided the proper folding sequences.  According to the other topologies tested in figure7, as well as the trRosetta predicted outcomes, there should be really interesting alpha/beta fold proteins in this set, but unfortunately the authors did not have crystal structures for those to really define the usefulness of the GM. 

We disagree that data in figure 7 indicate a problem with the yeast expression assay. As we now note in lines 414-419, “Five of the eight monomeric/oligomeric designs are highly thermostable (S24 Fig); four do not unfold even at a temperature of 99C. These four are precisely the non-aggregating proteins with stability score > 1, providing additional experimental validation to the previously published finding [23] that stability scores are reasonably correlated with thermal stability.”

As we note in line 420, both the Reverse GM and trRosetta predict a three-helix structure for the crystallized protein. While the input to the GM requested a four-helix structure, the GM is a stochastic model, and especially when considering results with low homology to the training set may not always give you a protein with the secondary sequence that was requested (we have highlighted that this matching of secondary structure is only probabilistic in lines 272-274).

We are as disappointed as anyone that we were able to obtain a crystal structure for only one of the designs.

All that said, as previously noted, the main utility of the GM at this point is to rapidly generate stable or stabilizable mini-proteins. We make no claims about the practical utility of doing so.

(3) The heroic experimental efforts the authors took to complete this study is unfortunately not very informative because of the black-box nature of the neural networks. In Rocklin et al., the authors used big-data to derive physical features that are relevant to making protein designs. This is not replicated in this paper because we don't know what neural networks learned. 

I am convinced that the experiments are well executed and model trainings are carefully done. There are some minor points that the authors can potentially clarify about their dataset but from what I can tell, both the EM and GM models are very carefully trained. All necessary controls are considered. 

I think this study would be much more interesting if the authors can somehow analyze what the EM model had learned from the assay.  The improvements to protease resistance seems very real and highly correlated with the model's predictions. The authors conducted a number of experiments to address potential over-fitting, and admittedly the model may still be overfitted.  But if the EM performance is truly predictive, maybe through this study they can really improve the yeast protease assay to really reveal what that assay is measuring, instead of using the assay as the gold standard for stability and try to make sequences to fit.

We agree that the inscrutability of neural networks is far from ideal in terms of learning explicable principles of protein design. One difficulty with applying network interpretation techniques to the EM is that, while we have strong intuitions about what a good image or a good sentence (the usual domains in which such techniques are deployed) looks like and how to interpret their constituent parts, we have no such intuition in the domain of protein design. One could regress against simple biophysical qualities as was done in Rocklin et al. (2017), but (a) that was already done, and (b) that limits you to simple insights regarding the simple biophysical qualities that you have identified to regress against.

I really don't see the value of the GM. There is no guarantee that the GM is really capturing the underlying distribution of viable sequences (perhaps the data are just too noisy, and the assumption that stability can be directly predicted from sequence might be wrong). 

As we note in lines 276-278, and as previously noted, the main utility of the GM at this point is to rapidly generate stable or stabilizable mini-proteins. We make no claims about the practical utility of doing so. Perhaps it will prove useful in the future, perhaps not.

Other issues:
(1) In the background, it is too dismissive of the role of protein 3D structures. The logic in the narrative is quite strange: (a) protein stability cannot be predicted from structure, (b) but there are all these other properties that people say you can predict from sequences, and (c) therefore we just dismiss all structure relevance and explore sequence only models. I think this is fundamentally flawed.  Especially that for (b), those properties are very narrowly defined, and it's really not generalizable to most proteins. 

We absolutely do not mean to dismiss 3D structure. Rather, in many cases, structure is not available, and we wanted to see if we could predict stability without it. We have attempted to clarify this.

(2) There are a few other neural-networks that generate sequences based on an input 3D backbone.  Perhaps it is good to mention them. Sequence generation is really not strictly "protein design"  For this paper, it might be more relevant because the sequences all correspond to some mini de novo proteins with an objective to improve stability, but in general there are too many papers claiming neural network "protein design" but are just glorified consensus generation. Historically protein designs are all based on structural models. 

There are absolutely tools that use neural networks (or biophysical constraints, or statistics of known natural proteins) to generate sequences to satisfy a backbone 3D structure. This is incredibly important work. It is not what we are doing, however.

(3) line 912, there is a missed symbol.

Thank you! Fixed.

(4) Figure2. Assuming that B and C are the same subset of clones, why don't their X-axis (predicted score) distributions match?

In B we excluded 281 outliers to avoid compressing the X axis too much. We made a note to this effect in the legend, thank you for catching this.

(5) For the data described in Methods, the designs used for training are split up by their topologies. But it is not clear to me if in building the test set, all similar topologies and sequence homologies are considered. That is, whether there is also a sequence level scrutiny in addition to just topology, such that when the authors mentioned data unseen by the model, it is really low sequence identity data.

As previously noted, we added some BLAST analysis to try to address this and related questions (lines 151-158).

(6)It looks like the reverse GM just serves the same function as psi-pred. How about just using Psi-pred?

We report the results of both the Reverse GM and PSIPRED in Fig. 7. We now also note (lines 279-281) that we are evaluating the ability of a language model to translate between primary and secondary sequence.

(7) page 15/16 Figure 7. It is not clear what's the rationale is behind the 12 clones chosen to be expressed recombinantly.  Are they based on stability score, random, etc.? 

We have made explicit our criteria in lines 398-400.

(8) Supplemental figures are not listed in order.

Thank you for noticing this (the paper has been through some substantial changes over the last few years)! We have fixed them.
